# Effects of Interferon-α/β on HBV Replication Determined by Viral Load

**DOI:** 10.1371/journal.ppat.1002159

**Published:** 2011-07-28

**Authors:** Yongjun Tian, Wen-ling Chen, Jing-hsiung James Ou

**Affiliations:** Department of Molecular Microbiology and Immunology, Keck School of Medicine, University of Southern California, Los Angeles, California, United States of America; Nationwide Children's Hospital, United States of America

## Abstract

Interferons α and β (IFN-α/β) are type I interferons produced by the host to control microbial infections. However, the use of IFN-α to treat hepatitis B virus (HBV) patients generated sustained response to only a minority of patients. By using HBV transgenic mice as a model and by using hydrodynamic injection to introduce HBV DNA into the mouse liver, we studied the effect of IFN-α/β on HBV *in vivo*. Interestingly, our results indicated that IFN-α/β could have opposite effects on HBV: they suppressed HBV replication when viral load was high and enhanced HBV replication when viral load was low. IFN-α/β apparently suppressed HBV replication via transcriptional and post-transcriptional regulations. In contrast, IFN-α/β enhanced viral replication by inducing the transcription factor HNF3γ and activating STAT3, which together stimulated HBV gene expression and replication. Further studies revealed an important role of IFN-α/β in stimulating viral growth and prolonging viremia when viral load is low. This use of an innate immune response to enhance its replication and persistence may represent a novel strategy that HBV uses to enhance its growth and spread in the early stage of viral infection when the viral level is low.

## Author Summary

Hepatitis B virus (HBV) is a major human pathogen that can cause severe liver diseases including hepatitis, liver cirrhosis and hepatocellular carcinoma. Approximately 350 million people worldwide are chronic carriers of this virus. Type I interferons (IFNs), which include IFN-α and IFN-β, are produced by the host to control microbial infections. However, the use of IFN-α to treat HBV patients has generated inconsistent results. By using mice as an animal model, we have investigated the effect of type I IFNs on HBV replication *in vivo*. Our results indicate that IFN-α/β can suppress HBV replication when viral load is high and enhance HBV replication when viral load is low. These effects of IFN-α/β on HBV are due in part to their abilities to regulate HBV gene expression. Our further studies reveal an important role of IFN-α/β in stimulating viral growth when viral load is low. This use of an innate immune response to enhance its replication may represent a novel mechanism that HBV uses to enhance its growth and spread in the early stage of infection when the viral level is still low.

## Introduction

Interferon-α (IFN-α) and interferon- β (IFN- β) are type I interferons, which are produced by the host in response to viral infections to inhibit viral replication [1]. After binding to its receptor, IFN-α/β activates the Janus kinase (JAK) and its downstream signal transducer and activator of transcription (STAT) and induces the expression of more than 300 IFN-stimulated genes (ISGs) and many antiviral proteins [2], [3]. IFN-α has been used to treat viral infections including hepatitis B virus (HBV), which chronically infects approximately 350 million people in the world. Unfortunately, IFN- α generates sustained virological response in only a minority of patients [4]. Little is known why the majority of HBV patients do not respond to the IFN-α therapy.

HBV is a small DNA virus that infects liver. Its genome is only 3.2 Kb in size and consists of four genes: the C gene codes for the viral core protein that forms the viral capsid and a related protein termed precore protein, which is the precursor of the secreted e antigen (HBeAg); the S gene codes for the viral envelope proteins, also known as surface antigens (HBsAg); the P gene codes for the viral DNA polymerase; and the X gene codes for a regulatory protein. To understand why IFN-α generates different responses in HBV patients, we studied the effect of IFN-α/β on HBV replication using mice as a model. Interestingly, we found that interferons could suppress HBV replication when viral load is high and enhance HBV replication when viral load is low. The suppression of HBV replication by IFN-α/β apparently involves both transcriptional and post-transcriptional regulations whereas the enhancement of HBV replication by IFN-α/β is mediated by transcription factors HNF3γ and STAT3. This use of type I interferons induced by its infection to enhance its replication thus represents a novel strategy that HBV may use to stimulate its growth and spread in the early stage of viral infection when the viral level is still low.

## Results

### Opposite effects of IFN-α/β on HBV replication in HBV transgenic mice

**Figure 1:**
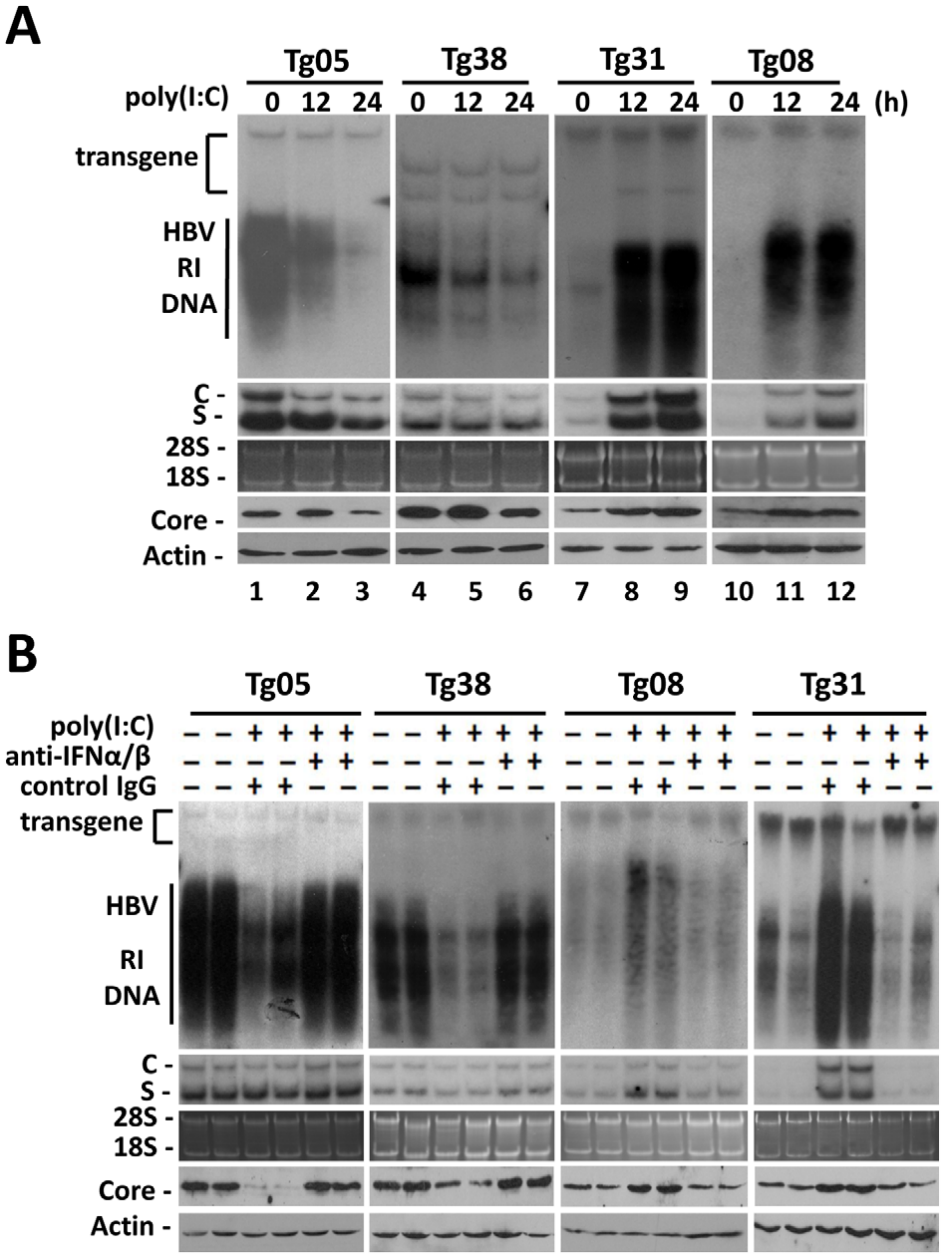
Effects of IFN- α/β on HBV in mice. (A) Effects of poly(I∶C) on HBV replication in transgenic mice. Four different HBV transgenic mouse lines were used for the studies. To minimize the variations of the results, mice of the same lineage were pre-screened for their serum HBV e antigen (HBeAg) levels, and only those with matched HBeAg levels were used for the studies. HBV transgenic mice were injected intravenously with saline and sacrificed 24 hours later (i.e., 0 hour poly(I∶C) treatment), or with 200 µg poly(I∶C) and sacrificed 12 hours or 24 hours later. Total liver DNA was isolated, digested with PvuII which does not cut into the HBV genome, and analyzed by Southern-blot for HBV DNA (top panel). The positions of HBV transgene, which served as the loading control for Southern-blot, and the HBV RI DNA are marked. Total liver RNA was also analyzed for HBV RNAs by Northern-blot (second panel from the top). C and S denote the HBV C gene and S gene RNA transcripts, respectively. For the RNA gel, 28S and 18S rRNAs were stained with ethidium bromide to serve as the loading control (middle panel). The liver homogenates were also used for Western-blot analysis for the HBV core protein (second panel from the bottom) and the β-actin (bottom panel). The latter served as the loading control. (B) Suppression of the effects of poly(I∶C) on HBV by anti-IFN-α/β antibodies in transgenic mice. HBV transgenic mice were injected with 250 µg control IgG or anti-IFN-α/β antibodies followed by injection with poly(I∶C). These mice were then sacrificed for the analysis of HBV DNA (top panel), HBV RNA (middle two panels), and the core protein and β-actin (bottom two panels). Two mice were used for each experiment for confirmation of the results.

We have previously produced four HBV transgenic mouse lines that carry either the wild type HBV genome (Tg05 and Tg08 mouse lines) or the mutated HBV genome that is incapable of expressing only the HBV X protein (HBx) (Tg31 and Tg38 lines) [5], which is a regulatory protein. These mouse lines contain replicating HBV DNA in the liver and produce mature viral particles in the blood (Figure S1A and S1B). To examine the possible effects of IFN-α/β on HBV *in vivo*, HBV transgenic mice were injected intravenously with the IFN-α/β inducer poly(I∶C), or with saline, which served as the control. Mice were sacrificed 12 hours or 24 hours after injection for the studies. The effect of poly(I∶C) on HBV DNA replication in the liver was analyzed by Southern blot. The level of HBV DNA replicative intermediates (RI) in the Tg05 mouse liver at 12 hours and 24 hours after poly(I∶C) injection was reduced by 54% and 80%, respectively (Figure 1A). When the HBV RNA was analyzed by Northern-blot, a slight reduction of the level by poly(I∶C) was also observed, particularly with the HBV C gene transcripts. When the HBV core protein was analyzed by Western-blot, its reduction by poly(I∶C) was inapparent at 12 hours, likely due to the stability of this protein. However, its reduction was apparent at 24 hours after injection (Figure 1A). For the Tg38 mouse line, the HBV DNA in the liver was also reduced in a time-dependent manner, although by a lesser degree (36% and 61% reduction at 12 hours and 24 hours, respectively, after injection). The reduction of the HBV RNA levels was not obvious. However, the core protein reduction was apparent at the 24-hour time point (Figure 1A). In contrast to Tg05 and Tg38 mouse lines, poly(I∶C) increased the HBV DNA, RNA and core protein levels in the liver of Tg31 and Tg08 mice (Figure 1A). These results indicated that poly(I∶C) could have different effects on HBV, depending on the mouse lines.

To determine whether the effect of poly(I∶C) on HBV was mediated by interferons, we first tested whether poly(I∶C) could indeed induce the interferon response in all four mouse lines. Total mouse liver RNA was isolated and analyzed for the expression of 2′-5′ oligoadenylate synthetase (2′,5′-OAS), a gene activated by IFN-α/β, by semi-quantitative reverse transcription PCR (RT-PCR). The expression of 2′,5′-OAS was indeed induced in the liver of all four mouse lines, indicating the induction of interferon response by poly(I∶C) (Figure S2A). We next injected HBV transgenic mice with antibodies directed against IFN-α/β one day prior to the injection of poly(I∶C). The administration of anti-IFN-α/β antibodies, but not the control antibody, abolished the induction of 2′,5′-OAS, indicating the ability of these anti-IFN-α/β antibodies to inhibit the activities of IFN-α/β in the liver (Figure S2B). Finally, we analyzed the effect of anti-IFN-α/β antibodies on HBV replication. The administration of anti-IFN-α/β antibodies, but not the control antibody, prevented poly(I∶C) from reducing the levels of HBV RI DNA, RNA and core protein in Tg05 and Tg38 mice and from increasing their levels in Tg08 and Tg31 mice (Figure 1B). If anti-IFN-α and anti-IFN-β antibodies were administered separately, the latter was found to be more efficient than the former in blocking the effect of poly(I∶C) (Figure S2C). This result might be due to the preferential induction of IFN-β by poly(I∶C) [6], or the difference in activities of these two cytokines [7]. We had also tested directly the role of IFN-α/β in the regulation of HBV replication by injecting Tg05 and Tg31 mice with IFN- α and IFN-β. Our results indicated that IFN- α had only a slight effect on HBV in these two mouse lines whereas IFN-β significantly suppressed HBV replication in Tg05 mice and enhanced HBV replication in Tg31 mice (Figure S2D).

In the studies shown in Figure 1, HBV transgenic mice were only treated with poly(I∶C) for up to 24 hours. To determine whether the effects of poly(I∶C) on HBV could persist, we analyzed its effects on HBV for one week. As shown in Figure S2E, the effects of poly(I∶C) on HBV could persist for one week, the endpoint of the analysis.

Our results thus indicated that the effect of IFN-α/β on HBV could vary depending on the mouse lines. This effect of IFN-α/β on HBV is independent of the HBx protein, as Tg05 and Tg08 mice carried the wild-type HBV genome and yet responded in opposite ways to IFN-α/β. Similarly, Tg31 and Tg38 carried the X-null HBV genome and also responded differently to IFN-α/β. However, there appeared to be a viral load-dependent effect, as IFN-α/β suppressed HBV replication in Tg05 and Tg38 mice, which produced higher levels of HBV, whereas they enhanced HBV replication in Tg08 and Tg31 mice, which produced lower levels of HBV (Figure S1A and S1B).

### Viral load-dependent effect of IFN-α/β on HBV replication

**Figure 2:**
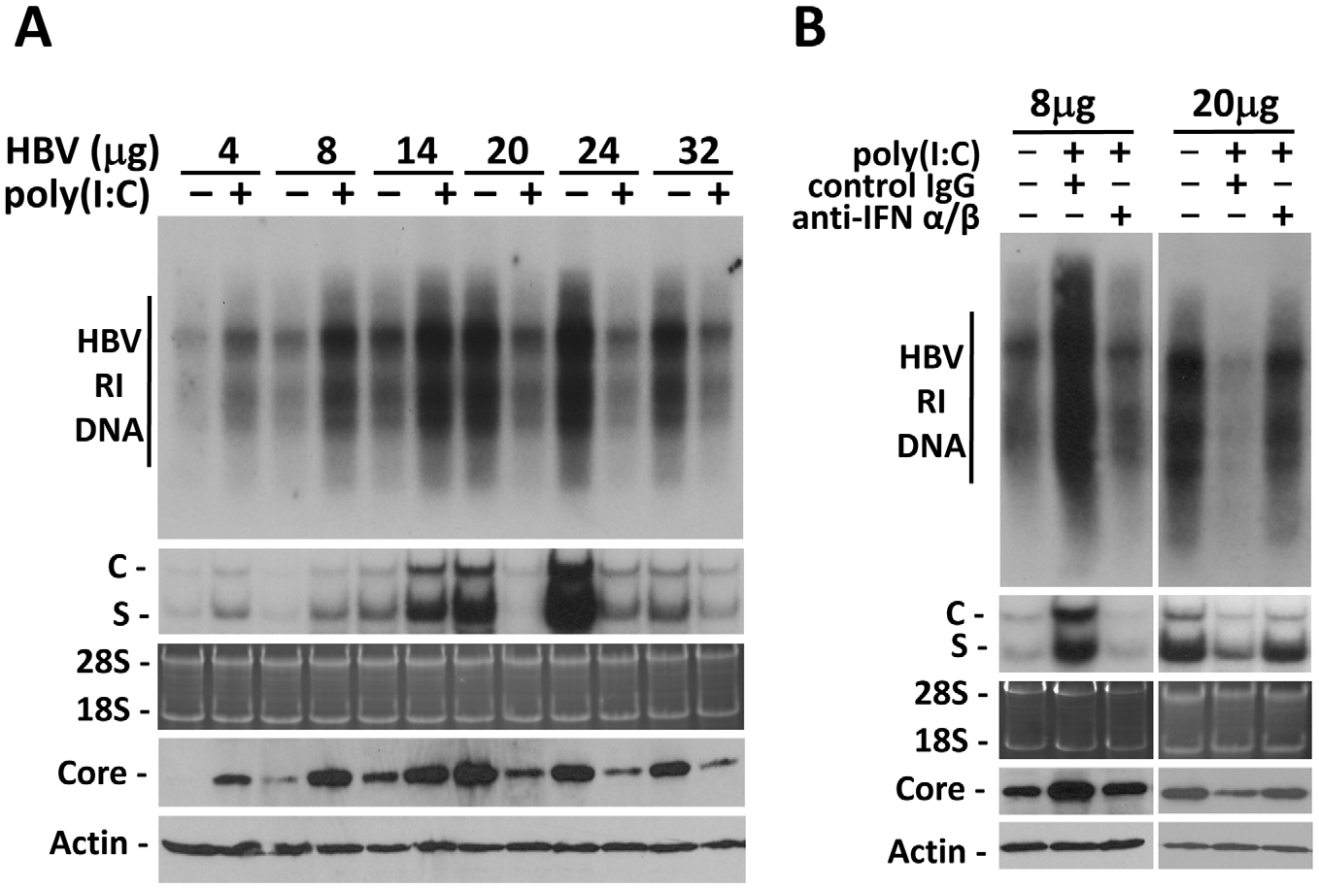
Viral load-dependent effect of IFN- α/β on HBV in mice. (A) Viral load-dependent effect of poly(I∶C) on HBV. Nine-week old, male naïve mice were hydrodynamically injected with 4, 8, 14, 20, 24 or 32 µg 1.3mer HBV genomic DNA via the tail vein. Three days after injection, mice with matched serum HBeAg levels in each group were selected and injected with saline or 200 µg poly(I∶C). All of the mice were sacrificed 4 days after the hydrodynamic injection. The levels of HBV RI DNA (top panel), RNA (middle two panels) and the core protein and β-actin (bottom two panels) were then analyzed. (B) Anti-IFN-α/β antibodies abolished the effects of poly(I∶C) on HBV in mice hydrodynamically injected with 1.3mer HBV DNA. Experiments were conducted as described in Figure 1B, with the exception that mice were injected with 8 µg or 20 µg 1.3mer HBV genomic DNA.

To examine whether the effect of IFN-α/β on HBV is indeed dependent on viral load, we performed the hydrodynamic injection, which is a rapid and convenient method for gene delivery into the mouse liver [8]. In this study, different amounts of the 1.3mer, over-length HBV DNA genome were injected via the tail vein into mice. Increasing the amount of HBV DNA in the injection led to an increasing level of HBV RI DNA, HBV RNA and the core protein in the liver until the amount of HBV DNA reached 24 µg (Figure 2A). Further increase of the HBV DNA amount to 32 µg for injection did not increase, but rather, decreased HBV RI DNA, HBV RNA and core protein levels in the mouse liver, perhaps due to the reduction in DNA delivery efficiency (Figure 2A). There was a positive correlation between the levels of HBV RI DNA in the liver and HBV titers in the sera (Figure S3). To test whether the effect of poly(I∶C) on HBV replication is dependent on viral load, mice were injected with ploy(I∶C) three days after the hydrodynamic injection of HBV DNA and sacrificed 24 hours later for HBV replication studies. Poly(I∶C) increased HBV DNA, RNA and core protein levels when the HBV DNA used for the injection was 4, 8 or 14 µg. However, poly(I∶C) decreased HBV DNA, RNA and core protein levels when the amount of HBV DNA used for the injection was 20, 24 or 32 µg (Figure 2A).

To determine whether the effect of poly(I∶C) on HBV in this hydrodynamic injection study was also mediated by IFN-α/β, we also injected mice with either the control antibody or the anti-IFN-α/β antibodies. The control IgG had no effect on the increase of HBV DNA, RNA and core protein levels induced by poly(I∶C) when mice were injected with 8 µg HBV DNA. However, this increase was abolished by anti-IFN-α/β antibodies. Similarly, although the control IgG had no effect on the decrease of HBV DNA, RNA and core protein levels by poly(I∶C) when mice were injected with 20 µg HBV DNA, anti-IFN-α/β antibodies diminished the suppressing effect of poly(I∶C) on HBV (Figure 2B).

The results indicate that the effect of IFN-α/β on HBV is dependent on viral load. They enhance HBV replication when viral load in the serum is low and suppress HBV replication when viral load is high. The viral DNA level in the serum that separates these two opposite responses appears to be in the vicinity of 10^7^ copies/ml (Figure S3), as the HBV replication was stimulated by interferons when the viral DNA level in the serum was lower than 10^7^ copies/ml whereas it was suppressed when the viral DNA level was higher than 10^7^ (Figure 2A and Figure S3).

### Activation of the HBV enhancer I/X promoter complex in mice by poly(I∶C)

**Figure 3:**
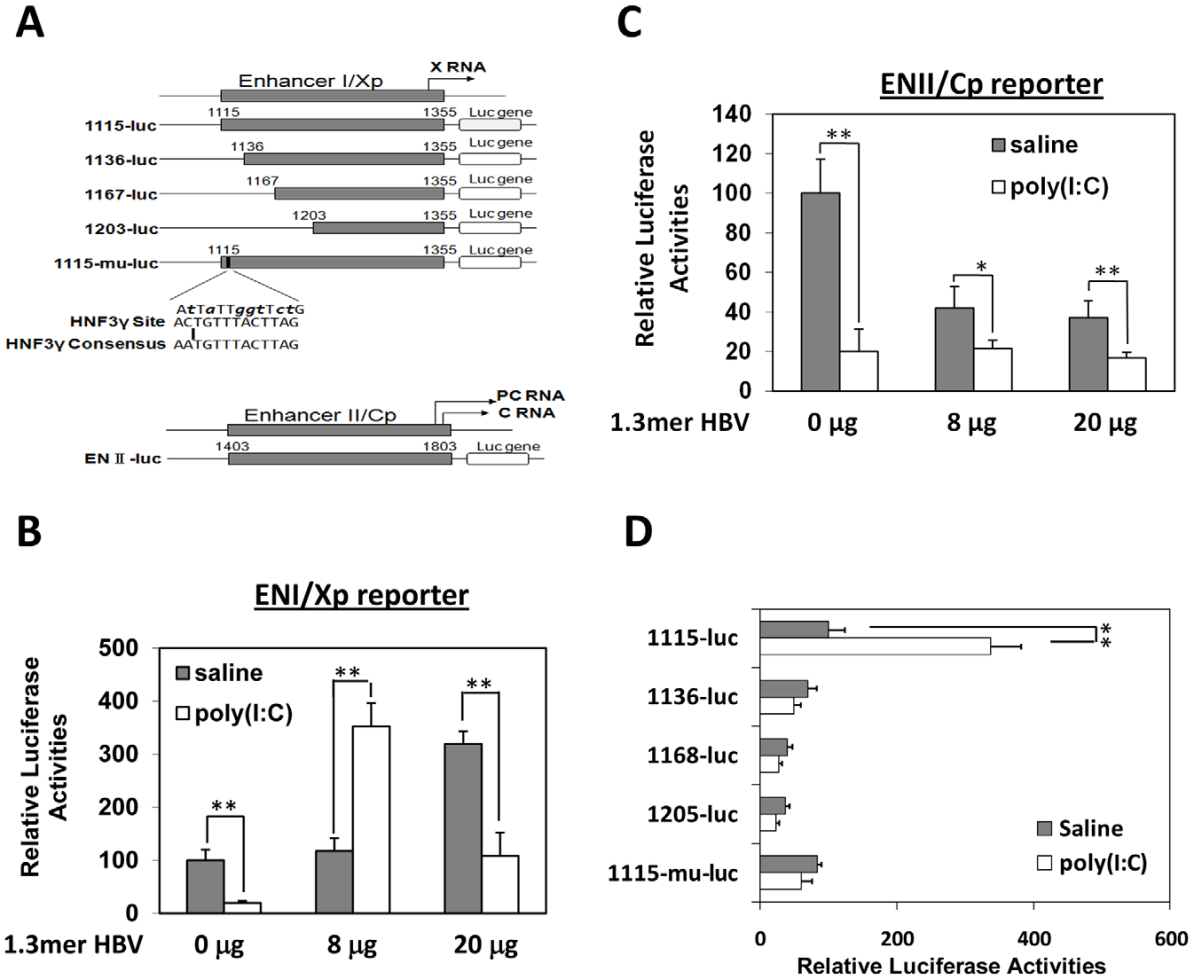
Effects of poly(I∶C) on HBV ENI/Xp and ENII/Cp in mice. (A) Illustration of the reporter constructs. The HNF3 binding sequence in the ENI enhancer and the consensus HNF3 binding sequence are also shown. The mutations introduced into 1115-mu-luc are shown in boldface lower-case letters. Arrows indicate the transcription start sites of the X RNA and the precore protein (PC) RNA. (B) Analysis of the ENI/Xp activity. pGL-3-1115-luc, which contains the HBV ENI/Xp complex that was linked to the luciferase reporter, was co-delivered with 0, 8 or 20 µg 1.3mer HBV into the mouse liver by hydrodynamic injection. A renilla luciferase reporter construct pRL-SV40 was also included in the injection to monitor the transfection efficiency. In all the experiments, if it is necessary, the vector control was included in the injection to ensure that the total amount of DNA used for the injection was the same between different samples. Forty-two hours after injection, mice were treated with saline (grey bar) or poly(I∶C) (empty bar) and sacrificed six hours later for the isolation of liver for the dual luciferase assay. The firefly luciferase activity was normalized against the renilla luciferase activity, which was not significantly affected by poly(I∶C). The firefly luciferase activity in the absence of 1.3mer HBV DNA and poly(I∶C) was arbitrarily defined as 100%. The results represent the mean±S.D. of three independent experiments. (C) Analysis of the ENII/Cp activity. Experiments were conducted as described in (B), with the exception that pGL-3-ENII-luc was used as the reporter. (D) Deletion-mapping analysis of the ENI/Xp complex. The reporter constructs were co-injected with 8 µg 1.3mer HBV DNA into mice. The experiments were conducted as described in (B). *, *p*<0.05; **, *p*<0.01.

Previous studies indicated that IFN-α/β suppressed HBV replication in transgenic mice that produced high levels of HBV by inhibiting the assembly of the viral capsid or by accelerating their degradation [9]. Our poly(I∶C) injection results indicated that IFN-α/β could also affect HBV RNA transcription or stability, as the reduction of HBV RNA levels in the liver of mice that produced high levels of HBV was also apparent after the injection of poly(I∶C) (e.g., Figure 2A and 2B). However, in mice that produced a low level of HBV, IFN-α/β significantly increased the levels of both HBV C gene and S gene RNA transcripts, suggesting that IFN-α/β might enhance HBV replication in these mice by enhancing the transcription of HBV genes, possibly by acting on the two HBV enhancers, which have global effects on HBV gene expressions [10]. To test this possibility, HBV DNA fragments containing enhancer I and its overlapping X promoter (ENI/Xp) or enhancer II and its overlapping C promoter (ENII/Cp) were linked to the firefly luciferase reporter (Figure 3A). These DNA constructs were delivered together with the 1.3mer HBV genomic DNA into the mouse liver by hydrodynamic injection. Poly(I∶C) decreased the expression of the firefly luciferase approximately three-fold when the ENI/Xp reporter construct was co-injected with 0 or 20 µg 1.3mer HBV genomic DNA. However, it increased the expression level of the luciferase three to four-fold when the reporter construct was co-injected with 8 µg 1.3mer HBV genome (Figure 3B). In contrast, poly(I∶C) reduced the expression level of the luciferase reporter from the ENII/Cp construct, regardless of whether this reporter construct was co-injected with 0, 8 or 20 µg HBV genomic DNA (Figure 3C). These results indicated that poly(I∶C) most likely activated HBV gene expression when the HBV DNA level was low by activating the ENI/Xp complex. Since poly(I∶C) could not activate the ENI/Xp complex in the absence of HBV genome (Figure 3B), this result also indicated an essential role of a low HBV genomic DNA level for poly(I∶C) to exert its enhancing effect. Note that, in the absence of poly(I∶C), the expression level of luciferase from the ENI/Xp reporter construct was higher in the presence of 20 µg HBV genomic DNA, and its expression level from the ENII/Cp reporter construct was higher in the absence of HBV DNA. The reason for these differences is unclear, but it might be related to the activities of HBV gene products on ENI/Xp and ENII/Cp complexes [11], [12], [13].

The ENI/Xp complex consists of multiple transcription factor binding sites [10], [14]. To further identify the IFN-α/β responsive element in this complex, we conducted the deletion-mapping experiments. The deletion of the sequence upstream of nt. 1136 was sufficient to abolish the stimulatory effect of poly(I∶C) on the ENI/Xp complex (Figure 3D), suggesting that the IFN-α/β responsive element resides at nt. 1115–1136. This sequence has previously been shown to contain HNF3 and STAT3 transcription factor binding sites [15], [16], [17]. To test whether the HNF3 binding site was indeed the IFN-α/β responsive element, we introduced mutations, which have previously been shown to abolish the HNF3 binding [15], into the ENI enhancer (Figure 3A). These mutations indeed abolished the response of ENI/Xp to IFN-α/β, confirming the role of the HNF3 binding site in mediating the effect of IFN-α/β (Figure 3D).

### Induction of HNF3γ by IFN-α/β to stimulate HBV gene expression and replication in mice with low HBV levels

**Figure 4:**
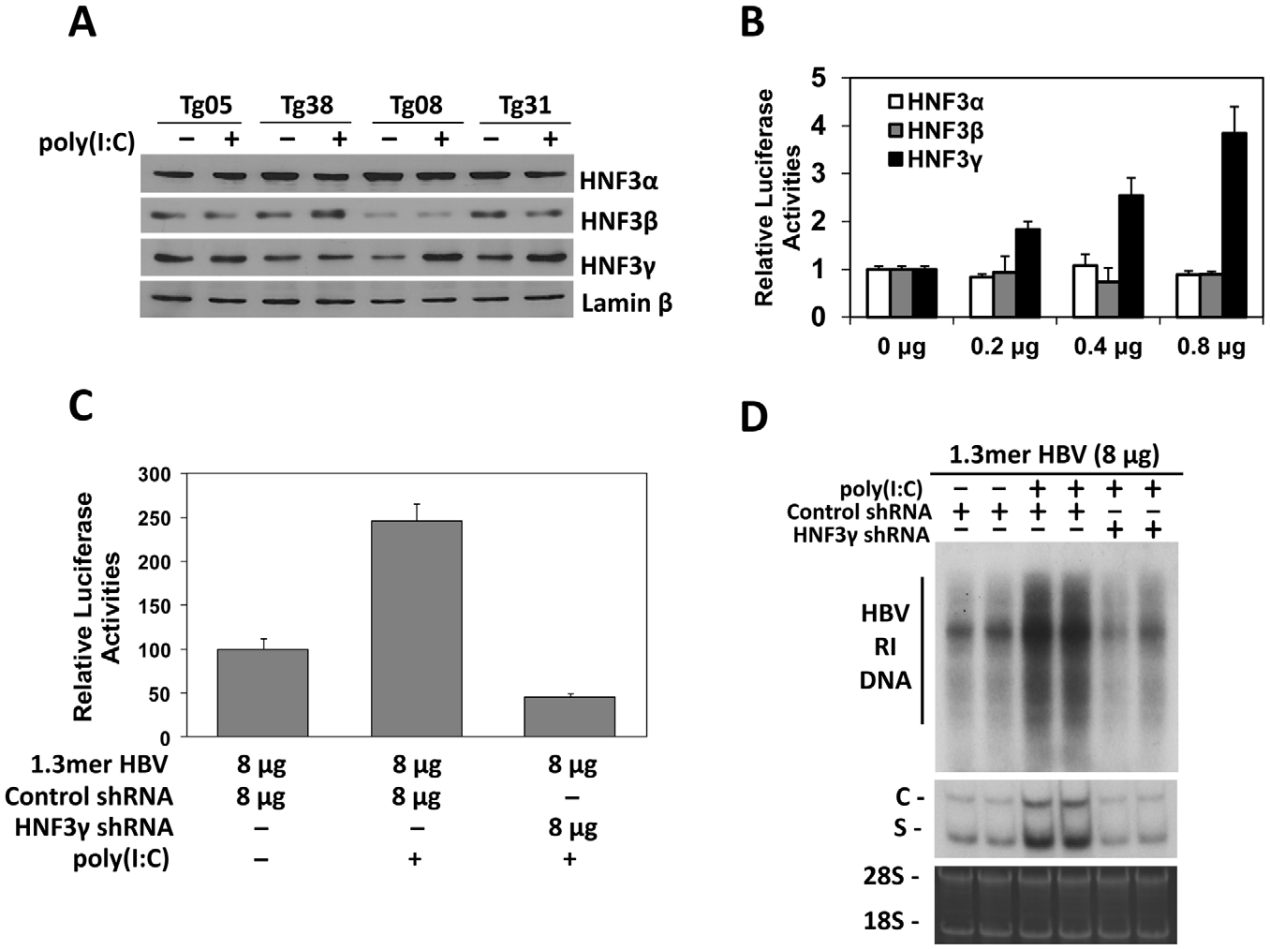
Effects of HNF3 on HBV replication. (A) Western-blot analysis for the expression of HNF3 isoforms. Liver nuclear extracts of HBV transgenic mice with (+) or without (−) poly(I∶C) injection were analyzed for HNF3α, HNF3β, HNF3γ, and lamin-β using their respective antibodies. (B) Effects of HNF3α, HNF3β and HNF3γ on HBV ENI/Xp activities in Huh7 cells. The ENI/Xp reporter construct pGL-3-1115-luc as well as the renilla luciferase reporter was co-transfected with the indicated amounts of the expression plasmid for HNF3α, HNF3β or HNF3γ into Huh7 cells. The control expression vector pRc/CMV was used to ensure that the total amount of DNA used in the transfection was the same among different experiments. The luciferase activities were determined as described in the Figure 3 legend. (C) Effects of HNF3γ on the ENI/Xp complex in mice. Nine-week old naïve mice were hydrodynamically injected with the ENI/Xp reporter construct, 8 µg 1.3mer HBV and the expression plasmid for either the control shRNA or the HNF3γ shRNA. Forty-two hours later, mice were further injected with poly(I∶C) or saline. Mice were sacrificed six hours later for the isolation of liver for the dual luciferase assay. Values represent means±S.D. of three independent experiments. The reporter activity in the absence of poly(I∶C) was arbitrarily defined as 100%. (D) Effects of HNF3γ on HBV replication in mice. Naïve mice were co-injected with 8 µg 1.3mer HBV and the expression plasmid for the control shRNA or HNF3γ shRNA, treated with saline or poly(I∶C) and sacrificed for the isolation of liver for HBV DNA and RNA analyses. Two mice were analyzed for each experiment for verification of the results.

There are three isoforms of HNF3, which are HNF3α, HNF3β and HNF3γ. All these three isoforms could bind to the HNF3 site in the HBV ENI enhancer [15]. To determine whether the expression of these three HNF3 isoforms was affected by poly(I∶C), we conducted the Western-blot analysis on the nuclear extracts of the mouse liver. While poly(I∶C) had no effect on the levels of HNF3α, HNF3β and the control lamin-β protein in the liver of all four of our HBV transgenic mouse lines, poly(I∶C) specifically increased the level of HNF3γ in Tg08 and Tg31 mice but not in Tg05 and Tg38 mice (Figure 4A). To further study the role of these three different HNF3 isoforms on the HBV ENI enhancer activity, the expression plasmids for these three different isoforms were separately co-transfected with the ENI/Xp reporter into Huh7 cells, a human hepatoma cell line. The over-expression of HNF3γ, but not HNF3α or HNF3β, could enhance the ENI/Xp activity in a dose-dependent manner (Figure 4B).

The results suggested that IFN-α/β might induce the expression of HNF3γ to activate the ENI enhancer in mice with a low HBV level. To test this possibility, we decided to use the shRNA to suppress the expression of HNF3γ. We first verified that the HNF3γ shRNA could indeed suppress the expression of HNF3γ and reduce the ENI/Xp activity in Huh7 cells (Figure S4). We next co-delivered the expression plasmid of this HNF3γ shRNA with the ENI/Xp reporter construct and 8 µg 1.3mer HBV DNA into the mouse liver by hydrodynamic injection. Poly(I∶C) could activate the HBV ENI/Xp complex in the presence of the control shRNA but not in the presence of the HNF3γ shRNA (Figure 4C). Similarly, the activation effect of poly(I∶C) on HBV DNA replication and RNA transcription was also abolished by the HNF3γ shRNA in mice injected with 8 µg 1.3mer HBV DNA (Figure 4D). These results demonstrated that the enhancing effect of IFN-α/β on HBV replication was mediated by HNF3γ.

### Activation of STAT3 by IFN-α/β to stimulate HBV gene expression and replication in mice with low HBV levels

**Figure 5:**
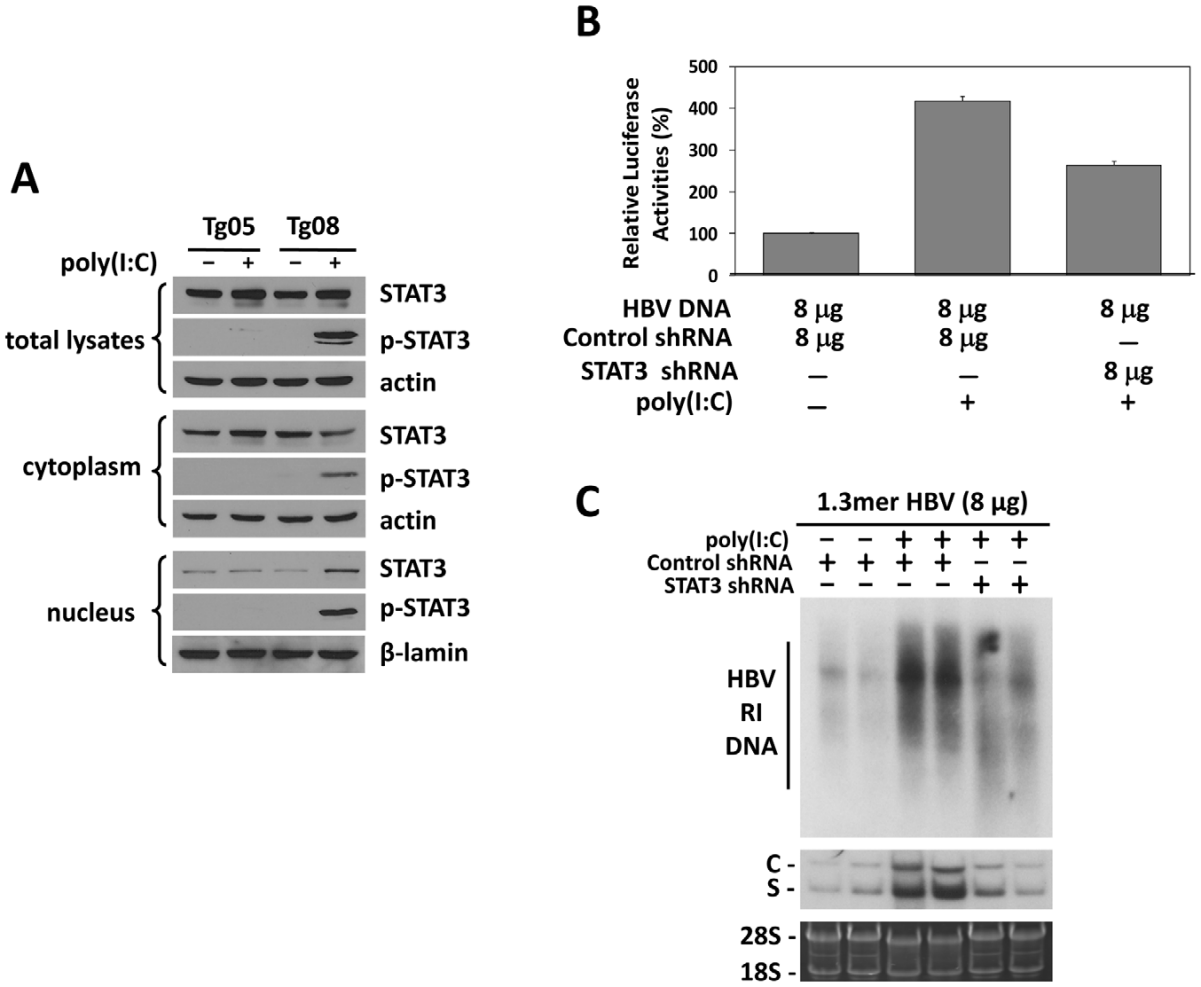
Analysis of STAT3 in HBV mice. (A) Western-blot analysis of STAT3 in HBV transgenic mice. Tg05 or Tg08 HBV transgenic mice with (+) or without (−) poly(I∶C) injection were sacrificed and the liver was isolated for Western-blot analysis. (B) Effect of STAT3 on the ENI/Xp activity in mice. Experiments were conducted as described in the Figure 4C legend, with the exception that the expression plasmid for HNF3γ shRNA was replaced with that for STAT3 shRNA. (C) Effects of STAT3 on HBV replication in mice. Similar to (B), experiments were conducted as described in the Figure 4D legend, with the exception that the expression plasmid for HNF3γ shRNA was replaced with that for STAT3 shRNA.

Previous studies indicated that HNF3 and STAT3 could bind to each other and cooperatively stimulate the HBV ENI enhancer [17]. Our finding that IFN-α/β induced the expression of HNF3γ to stimulate the HBV ENI enhancer prompted us to examine whether IFN-α/β also affects STAT3 in mice with a low HBV level. Although poly(I∶C) had no effect on STAT3 in Tg05 mice that produced a high level of HBV, it activated STAT3 in Tg08 mice, which produced a low level of HBV, as evidenced by the increased level of phosphorylated STAT3 (p-STAT3) and its association with the nuclear fraction (Figure 5A). To further determine the role of STAT3 in mediating the effect of IFN-α/β on HBV gene expression, we also conducted the shRNA-knockdown experiment to suppress the expression of STAT3. We first analyzed the effect of the STAT3 shRNA and demonstrated that it could reduce the expression level of STAT3 by approximately 40% and a similar level of the ENI/Xp activity in a reporter assay in Huh7 cells (Figure S5). We then injected mice with the expression plasmid for the STAT3 shRNA or a control shRNA, the ENI/Xp reporter construct and 8 µg HBV genomic DNA, and analyzed the effect of poly(I∶C) on the ENI/Xp activity. The STAT3 shRNA reduced the activation effect of poly(I∶C) on the ENI/Xp complex (Figure 5B). Similar to the HNF3γ results, the STAT3 shRNA, but not the control shRNA, also partially reduced the enhancing effect of poly(I∶C) on HBV DNA replication and RNA transcription (Figure 5C). These results indicated that STAT3 also played an important role in mediating the enhancing effect of IFN-α/β on HBV gene expression. The lack of complete inhibition of the effects of poly(I∶C) by the STAT3 shRNA was probably due to the inefficiency of this shRNA to knockdown the expression of STAT3 (Figure S5).

### Kinetics of HBV replication in mice injected with high and low doses of HBV DNA

**Figure 6:**
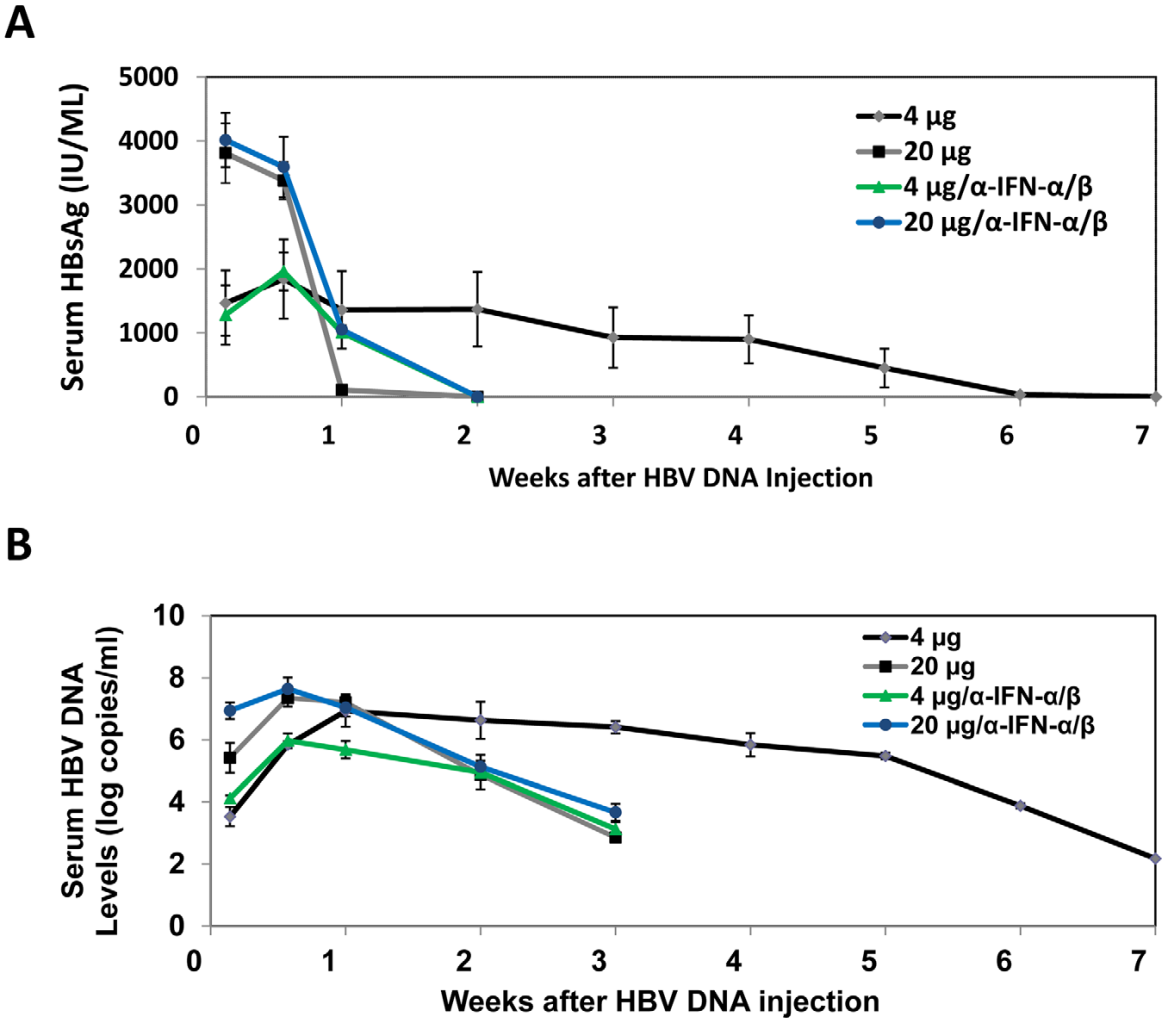
Analysis of HBV replication in mice. The sera of eight mice injected with 4 µg 1.3mer HBV DNA, six mice injected with 20 µg HBV DNA, 3 mice co-injected with 20 µg 1.3mer HBV DNA and anti-IFN-α/β antibodies, and 3 mice co-injected with 4 µg 1.3mer HBV DNA and anti-IFN-α/β antibodies, were collected at the time points indicated and analyzed for HBsAg by ELISA (A) and HBV DNA by real-time PCR (B). For mice treated with anti-IFN-α/β antibodies, 250 µg antibodies were injected immediately after DNA injection and thereafter on a weekly basis. The results shown in (B) represent the mean±S.D. of all of the mice used in each group. The results shown in (A) represent the mean±S.D. of the mice that were still positive for HBsAg at that particular time point.

The observation that the effect of IFN-α/β on HBV is dependent on viral load prompted us to investigate how that effect may affect viral growth *in vivo*. We injected mice with either 4 µg or 20 µg1.3mer HBV genomic DNA and analyzed HBV surface antigen (HBsAg) and DNA levels in the mouse serum over a seven-week period of time. Although mice injected with 20 µg HBV DNA produced initially a higher serum level of HBsAg, this antigen became undetectable after a week. In contrast, mice injected with 4 µg HBV DNA produced a lower-level of surface antigen that persisted for well over a month (Figure 6A). Importantly, this prolonged antigenemia was abolished if mice injected with 4 µg HBV DNA were also injected with anti-IFN-α/β antibodies on a weekly basis, indicating a role of IFN-α/β in maintaining antigenemia. In contrast, although anti-IFN-α/β antibodies slightly increased the level of HBsAg in mice injected with 20 µg HBV DNA one week after DNA injection, they did not prolong antigenemia, indicating the possible involvement of other factors in limiting viral persistence. Since HBsAg could be masked by the antibodies that it elicited, we also analyzed the serum HBV DNA levels. Mice injected with 20 µg HBV DNA produced a higher level of serum HBV DNA than mice injected with 4 µg HBV DNA within the first four days after injection. However, this difference was not observed one week after injection. Furthermore, the serum HBV DNA level of mice injected with 20 µg DNA declined rapidly thereafter and became undetectable after three weeks, whereas this serum DNA level persisted for a much longer period of time in mice injected with 4 µg DNA (Figure 6B). Similarly, if mice injected with 4 µg HBV DNA were also injected with anti-IFN- α/β antibodies, their serum viral DNA level also became undetectable after three weeks. The anti-IFN-α/β antibodies had little effect on the serum HBV DNA level in mice injected with 20 µg HBV DNA, except at the earliest time point. When the alanine aminotransferase (ALT) was analyzed to monitor liver injury, high levels of ALT were observed only within the first few days, most likely caused by the hydrodynamic injection, which causes liver injury (Figure S6). The ALT levels were low after that, indicating minimal liver injuries. These results indicated that the low-dose inoculation of the HBV DNA could lead to a more persistent viral replication and this persistence was dependent on IFN-α/β.

## Discussion

**Figure 7:**
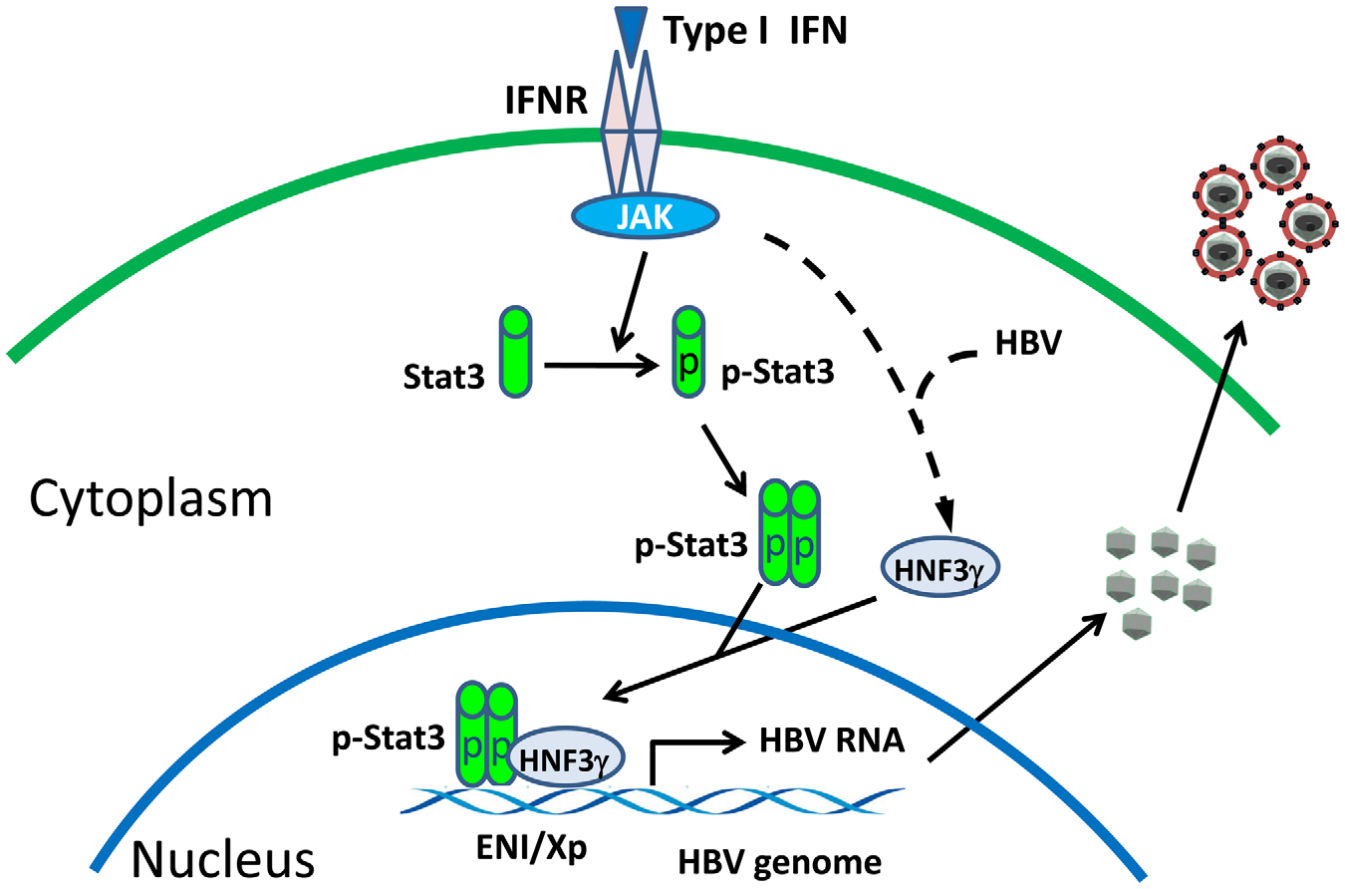
Mechanism of the activation of HBV replication by IFN- α/β when the HBV level is low. IFNR, interferon receptor; JAK, Janus kinase; p-Stat3, phosphorylated Stat3.

Interferons are thought to play an important role in the control of viral infections. Indeed, previous studies using transgenic mice that produced a high level of HBV indicated that interferons could suppress HBV replication [18]. Our observation that IFN-α/β could enhance HBV replication in mice that produced a low level of HBV is thus rather intriguing. Our results indicated that this enhancement was due to the activation of the HNF3γ gene and STAT3, which then stimulate the HBV ENI enhancer activity. The induction of HNF3γ by IFN-α/β requires the presence of a low level of HBV, as such induction was not observed in the presence of a high level of HBV DNA (Figure 4A) or in naïve mice (data not shown). STAT3 is activated in the presence of a low level of HBV, but it was not activated in the presence of a high level of HBV (Figure 5A). Since STAT3 can also be activated by IFN- α/β in hepatocytes in the absence of HBV [19], [20], it appears that HBV at a high replication level can prevent the activation of STAT3 by IFN-α/β. These observations indicate an interesting interplay between HBV and the interferon signaling pathway. A model of how IFN-α/β enhances HBV replication in illustrated in Figure 7. In this model, the binding of IFN-α/β to its receptor activates STAT3, likely due to the phosphorylation by JAK, which is associated with the IFN-α/β receptor and is activated upon binding of IFN-α/β to its receptor. In the mean time, the activated interferon signaling pathway also interacts with HBV to induce the expression of HNF3γ, which then binds cooperatively with STAT3 to the HBV ENI enhancer to stimulate HBV gene expression and viral replication. How HBV may interact with the JAK-STAT signaling pathway to induce the expression of HNF3γ is still not clear. This effect is independent of the HBx protein, since Tg31 mice carried the X-null HBV genome but yet HNF3γ could be induced by poly(I∶C) in this mouse line (Figure 4A). Clearly, if another HBV gene product such as that of the S, C or P gene is involved, this gene product must exert a dose-dependent effect since only a low replication level of HBV could induce HNF3γ.

The observation that IFN-α/β enhances HBV replication when the HBV DNA level is low may represent a mechanism by which HBV uses to establish its infection in patients, as the viral level is expected to be low in patients during the early stage of HBV infection. This possibility is supported by our observation that the injection of a small amount of HBV genomic DNA into mice could lead to prolonged viremia in an IFN-α/β-dependent manner (Figure 6). As our mouse model does not allow the reinfection of hepatocytes by HBV, it is conceivable that, if reinfection is possible, viral replication can persist in mice for an even longer period of time. Indeed, it has been shown that low-dose (1 or 10 genome-equivalent copies) inoculations of HBV into chimpanzees would lead to the spread of the virus and result in the infection of 100% of hepatocytes and prolonged immunopathology [21]. In contrast, the inoculation of between 10^4^ and 10^8^ genome copies of the virus led to a limited spread of the virus in the liver and the speedier clearance of the virus. Based on our findings described in this report, it is conceivable that the initial IFN-α/β response to the low-level HBV inoculation enhanced viral replication and spread and prolonged viral infection in chimpanzees.

Although the injection of IFN- α/β antibodies could suppress viral persistence in mice injected with 4 µg HBV DNA, the injection of IFN-α/β antibodies did not prolong viral persistence in mice injected with 20 µg HBV DNA. This result indicated that the removal of IFN-α/β alone was not sufficient to maintain viral persistence when viral load is high. The reason for this is unclear, but it may involve other factors such as additional cytokines (e.g., IFN-γ) that may be induced by high HBV load [18].

Recent studies indicated that the HBx protein could bind to MAVS (also known as IPS-1, VISA or Cardiff), which is an important adaptor molecular of the RIG-I signaling pathway, to suppress the induction of IFN-β [22], [23]. It has also been reported that the HBV DNA polymerase could bind to the DDX3 deadbox RNA helicase to suppress its interaction with TBK1/IKKε and the induction of IFN-β [24], [25]. In contrast to HBV structural proteins, HBx and the HBV polymerase are produced at a much lower level during viral replication. For this reason, it is likely that these two HBV products can only efficiently suppress the induction of type I interferons when the viral replication level is high. It is conceivable that the lag period before the effective concentrations of HBx and polymerase are reached in HBV-infected cells will allow HBV to use the interferon response to stimulate its gene expression and replication. However, once HBV has replicated to a high level and interferons become a negative regulator for HBV replication, HBx and the viral polymerase will exert their anti-interferon activities to enhance the survival of the virus.

IFN-α has been used to treat HBV patients, but it generated sustained response in only a minority of patients. Our observation that IFN-α/β could have opposite effects on HBV in a viral load-dependent manner indicates that viral load may be one of the reasons why this therapy has generated inconsistent responses in HBV patients.

## Materials and Methods

### Ethics statement

Our studies on mice were conducted in accordance with the recommendations in the *Guide for the Care and Use of Laboratory Animals* of the National Institutes of Health. Our animal protocol was approved by the Institutional Animal Care and Use Committee of the University of Southern California.

### HBV transgenic mice and DNA plasmids

HBV transgenic mouse lines Tg05 and Tg08 have been previously described [5], [26]. These two mouse lines carried the 1.3mer, over-length wild-type HBV genome. Tg31 and Tg 38 also carried 1.3mer HBV genome, with the exception that the expression of X protein was abolished by the introduction of an A-to-C mutation at nt.1377 to remove the initiation codon of the X protein and a C-to-T mutation at nt.1398 to introduce a premature termination codon in the X coding sequence. All of the experiments were conducted using age-matched male mice with the B6 genetic background.

The plasmid p1.3×HBV, which contains the 1.3mer over-length HBV genome, has been described before [27]. pCMV-HNF3α, pCMV-HNF3β and pCMV-HNF3γ, which express HNF3α, HNF3β and HNF3γ, respectively, have also been described [15]. The expression plasmids for mouse HNF3γ shRNA, STAT3 shRNA and control shRNA were purchased from Sigma-Aldrich. The HBV ENI/X reporter constructs pGL-3-1115-luc, pGL-3-1136-luc, pGL-3-1167-luc and pGL-3-1203-luc, which contained nt.1115–1355, 1136–1355, 1167–1355 and 1203–1355, respectively, of the HBV genome, were generated by PCR amplification of the HBV DNA fragment for cloning into the pGL3-basic vector (Promega). The plasmid pGL-3-ENII-luc was constructed by insertion the ENII/core promoter complex (nt.1403–1803) into the pGL3-basic vector. pRL-SV40 (Promega), which expresses the renilla luciferase, was included in the transfection studies to serve as the internal control to monitor the transfection efficiency.

### Injection with poy(I∶C)

Age and HBeAg-matched male HBV transgenic mice or naïve mice were injected intravenously with 200 µl saline with or without poly(I∶C) (200 µg/mouse). Mice were sacrificed 24 hours later. The serum was collected and the liver was harvested and stored at −80°C for analyses.

### Hydrodynamic injection of p1.3×HBV

Nine-week old male mice were injected via the tail vein with p1.3×HBV in 5–8 seconds in a volume of saline equivalent to 8% of the body weight of the mouse. In all the injection experiments, the vector DNA pUC19 was included if necessary to ensure that the total amount of DNA used for injection is identical among different mice. 24 hours after the hydrodynamic injection, the serum was collected and HBeAg was assayed by ELISA. Mice matched by body weight, age and HBeAg levels were used for injection with poly(I∶C).

### Antibodies

Rabbit anti-mouse IFN-α (PBL, New Jersey) and hamster anti-mouse IFN-β (Biolegend, San Diego) antibodies were used in this study. Purified rabbit IgG (Cell Signaling Tech.) and hamster IgG (Abcam) were used as the control antibodies. Rabbit anti-HNF3α (Abcam), anti-HNF3β (Cell Signaling Tech.), anti-HNF3γ (Sigma–Aldrich Co.), anti-STAT3 (Cell Signaling Tech.), anti-phosph-STAT3(Tyr705) (Cell Signaling Tech.) and anti-lamin-β (Abcam) antibodies were used for Western-blot.

### Southern-, Northern- and Western-blot analyses

Liver tissues were homogenized in DNA lysis buffer (20 mM Tris-HCl, pH 7.0, 20 mM EDTA, 50 mM NaCl, 0.5% SDS), incubated for 16 hours at 37°C with proteinase K (600 µg/ml) and then phenol/chloroform extracted for the isolation of DNA. The HBV RI DNA in the core particles was isolated using our previous protocol [27]. For RNA isolation, liver tissues were homogenized in Trizol (Invitrogen) and total RNA was isolated following the manufacturer's protocol. Both Southern and Northern blot analyses were conducted using the ^32^P-labeled HBV DNA probe. For Western-blot analysis, liver tissues were homogenized in the RIPA solution (10 mM Tris-HCl, pH 7.0, 150 mM NaCl, 1% Triton X-100, 1% sodium deoxycholate, and 0.1% sodium dodecyl sulfate) and, after a brief centrifugation to remove cell debris, the protein concentrations were determined by Bradford BCA (Biorad) and the Western blot was performed using our previous procedures [28].

### Real-time PCR analysis of serum HBV DNA

10 µl mouse serum was added into 100 µl lysis buffer (20 mM Tris-HCl, 20 mM EDTA, 50 mM NaCl, and 0.5%SDS) containing 27 µg proteinase K. After incubation at 65°C overnight, viral DNA was isolated by phenol/chloroform extraction and ethanol precipitation. The DNA pellet was rinsed with 70% ethanol and resuspended in 10 µl TE (10 mM Tris-HCl [pH 7.0], 1 mM EDTA). For hydrodynamic injection studies, 10 µl serums was digested with 10 µg DNase I and micrococcal nuclease for 30 min at 37°C to remove free DNA. HBV DNA was then isolated as described above. For HBV real-time PCR analysis, the following primers were used: forward primer, 1552-CCGTCTGTGCCTTCTCATCTG-1572; and reverse primer, 1667-AGTCCTCTTATGTAAGACCTT-1646. The TaqMan probe used was 1578-CCGTGTGCACTTCGCTTCACCTCTGC-1603. The assays were performed as described [29].

### The luciferase reporter assay

The reporter constructs were delivered into the mouse liver by hydrodynamic injection. Nine-week old male mice were used in the studies. Twenty-four hours after injection, the serum was collected and HBeAg was assayed by ELISA. Mice with matched body weight and the HBeAg level were injected intravenously with 200 µl saline with or without poly(I∶C) (200 µg/mouse) at 42 hours after hydrodynamic injection and sacrificed 6 hours later. The mouse liver was isolated and stored at −80°C. The firefly luciferase and the renila luciferase activities were measured using the dual luciferase assay kit (Promega). The firefly luciferase activities were normalized against the renilla luciferase activities, which served as the internal control. All of the experiments were repeated at least three times.

### Serum ALT and ELISA assays

Serum ALT levels were measured using the ALT kit (Cayman Chemical Company, USA). HBsAg and HBeAg were measured using their respective ELISA kit (International Immuno-Diagnostics, CA). All of these assays were conducted following the manufacturers' instructions.

## Supporting Information

Figure S1 **Replication of HBV in transgenic mice. (A) HBV DNA and RNA levels in the mouse liver.** Top panel, ethidium bromide staining of the chromosomal DNA, which served as the loading control for Southern blot; second panel from the top, Southern-blot analysis of HBV DNA; third panel from the top, Northern-blot analysis of HBV RNA; bottom panel, ethidium bromide staining of the RNA gel to serve as the loading control. The HBV DNA replicative intermediates (RI) appeared as a smear on the gel. C and S indicate HBV C gene and S gene transcripts, respectively. The locations of 28S and 18S rRNAs are also indicated. **(B) HBV DNA levels in the sera of different transgenic mouse lines.** Nine-week old male HBV transgenic mice were used for the studies. HBV DNA was extracted from the serum and quantified by real-time PCR.
(TIF)

Figure S2 **Effects of poly(I∶C) on HBV transgenic mice.** **(A) Induction of 2′,5′-OAS in the mouse liver by poly(I∶C).** Total liver RNA was isolated from mice 24 hours after injection with saline (−) or poly(I∶C) (+) and analyzed by semi-quantitative RT-PCR for 2′,5′-OAS RNA and GAPDH RNA. The latter served as the control. **(B) Suppression of the interferon response in the mouse liver by the anti-IFN-α/β antibodies.** HBV transgenic mice were injected intravenously with the control IgG or anti-IFN-α/β antibodies (500 µg/mouse) and then with 200 µg poly(I∶C) 16 hours later. Mice were sacrificed 24 hours after the poly(I∶C) injection for the isolation of total liver RNA, which was used for semi-quantitative RT-PCR for the analysis of 2′,5′-OAS RNA and GAPDH RNA. Mice without the injection of poly(I∶C) and antibodies were also included in the studies to serve as the control. **(C) Suppression of poly(I∶C)-induced HBV replication in Tg31 HBV transgenic mice by anti-IFN-α/β antibodies.** Tg31 HBV transgenic mice were injected intravenously with the control IgG (lane 2), anti-IFN-α and anti-IFN-β antibodies together (lane 3), the anti-IFN-α antibody alone (lane 4), or the anti-IFN-β antibody alone (lane 5), followed by the injection with 200 µg poly(I∶C) 16 hours later (lanes 2–5). Mice were sacrificed 24 hours after the injection with poly(I∶C) for the analysis of HBV DNA (top panel), HBV RNA (middle two panels), and the core protein and β-actin (bottom two panels) in the liver. **(D) Effects of IFN-α/β on HBV replication in Tg05 and Tg31 mice.** Tg05 and Tg08 mice were injected with PBS, IFN- (1.4×10^5^ units) or IFN-β (1.6×10^5^ units) and sacrificed 24 hours later for the isolation of liver for analysis RNA (second panel from the top). The ribosomal RNAs (third panel from the top) and GAPDH RNA (bottom panel) were used as the loading control for Northern-blot analysis. **(E) Prolonged effect of poly(I∶C) on HBV replication.** Tg05 and Tg08 mice were injected with poly(I∶C) on a daily basis and sacrificed at the indicated time points for HBV DNA and RNA analysis as described in (D).
(TIF)

Figure S3 **Quantification of HBV titers in the sera of mice injected with HBV DNA.** Mice were injected with the indicated amount of the 1.3mer HBV DNA in phosphate-buffered saline (PBS). Four days later, mouse sera were treated with DNase I and micrococcal nuclease to remove free DNA. The HBV virion-associated DNA was then extracted and analyzed by real-time PCR. The results represent the average of at least three different mice.
(TIF)

Figure S4 **Effects of HNF3γ on the ENI/Xp activity in Huh7 cells.** Huh7 cells were co-transfected with the ENI/Xp reporter construct and the expression plasmid for the control shRNA or the HNF3γ shRNA for forty-eight hours and then lysed for Western-blot analysis for HNF3γ and lamin-β (top panel). The latter is a nuclear protein and served as the loading control. The plasmid pRL-SV40, which expresses renilla luciferase, was included in the transfection to monitor the transfection efficiency. Cell lysates were analyzed for the luciferase activities using the dual luciferase assay (Promega) (bottom panel).
(TIF)

Figure S5 **Effects of STAT3 on the ENI/Xp activity in Huh7 cells.** The experiments were conducted as described in the legend to Figure S4, with the exception that the HNF3γ shRNA was replaced with the STAT3 shRNA. Top panel, Western-blot analysis of STAT3 and α-actin; bottom panel, relative firefly luciferase activities.
(TIF)

Figure S6 **Analysis of ALT levels in mice injected with HBV DNA.** The serum samples collected from mice shown in Fig. 6 were analyzed for the ALT levels using the ELISA kit. The numerical ALT levels at individual time points are shown in the Table. N.D., not determined.
(TIF)
