# Supplementary figures and images for: Effects of Interferon-α/β on HBV Replication Determined by Viral Load
Source: PLoS Pathog. 2011 Jul 28;7(7):e1002159. doi: 10.1371/journal.ppat.1002159 (PMC3145790; doi:10.1371/journal.ppat.1002159)

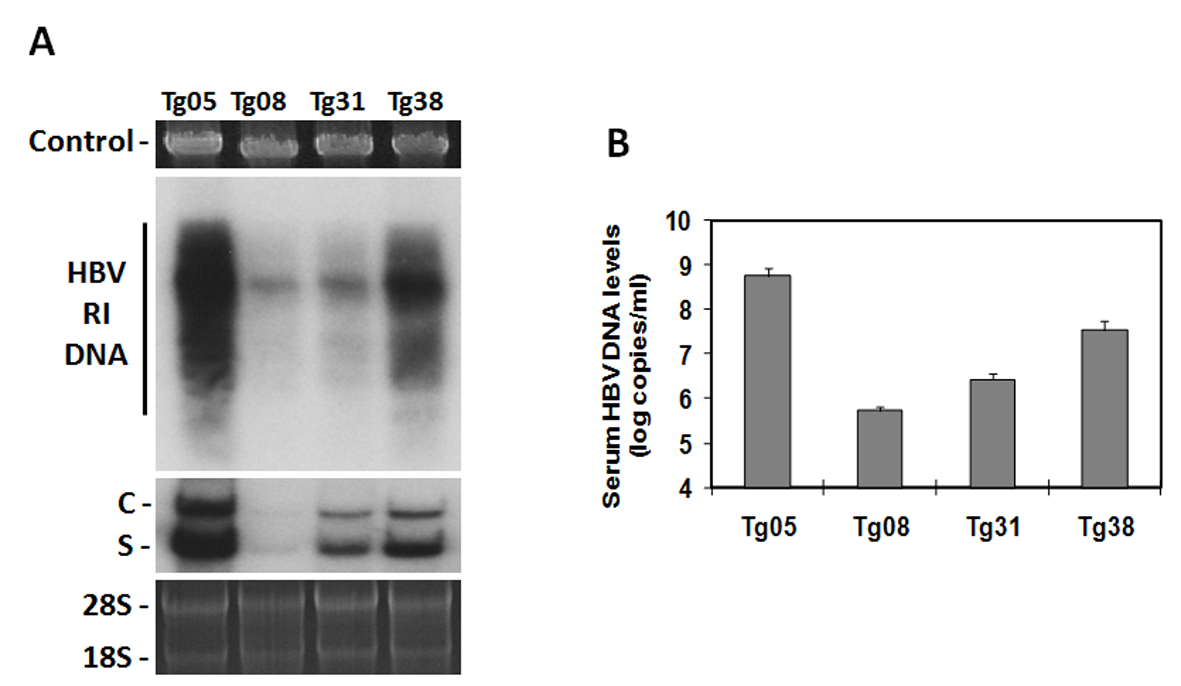

Supplement: Figure S1 — Replication of HBV in transgenic mice. (A) HBV DNA and RNA levels in the mouse liver. Top panel, ethidium bromide staining of the chromosomal DNA, which served as the loading control for Southern blot; second panel from the top, Southern-blot analysis of HBV DNA; third panel from the top, Northern-blot analysis of HBV RNA; bottom panel, ethidium bromide staining of the RNA gel to serve as the loading control. The HBV DNA replicative intermediates (RI) appeared as a smear on the gel. C and S indicate HBV C gene and S gene transcripts, respectively. The locations of 28S and 18S rRNAs are also indicated. (B) HBV DNA levels in the sera of different transgenic mouse lines. Nine-week old male HBV transgenic mice were used for the studies. HBV DNA was extracted from the serum and quantified by real-time PCR. (TIF) [file ppat.1002159.s001.tif]

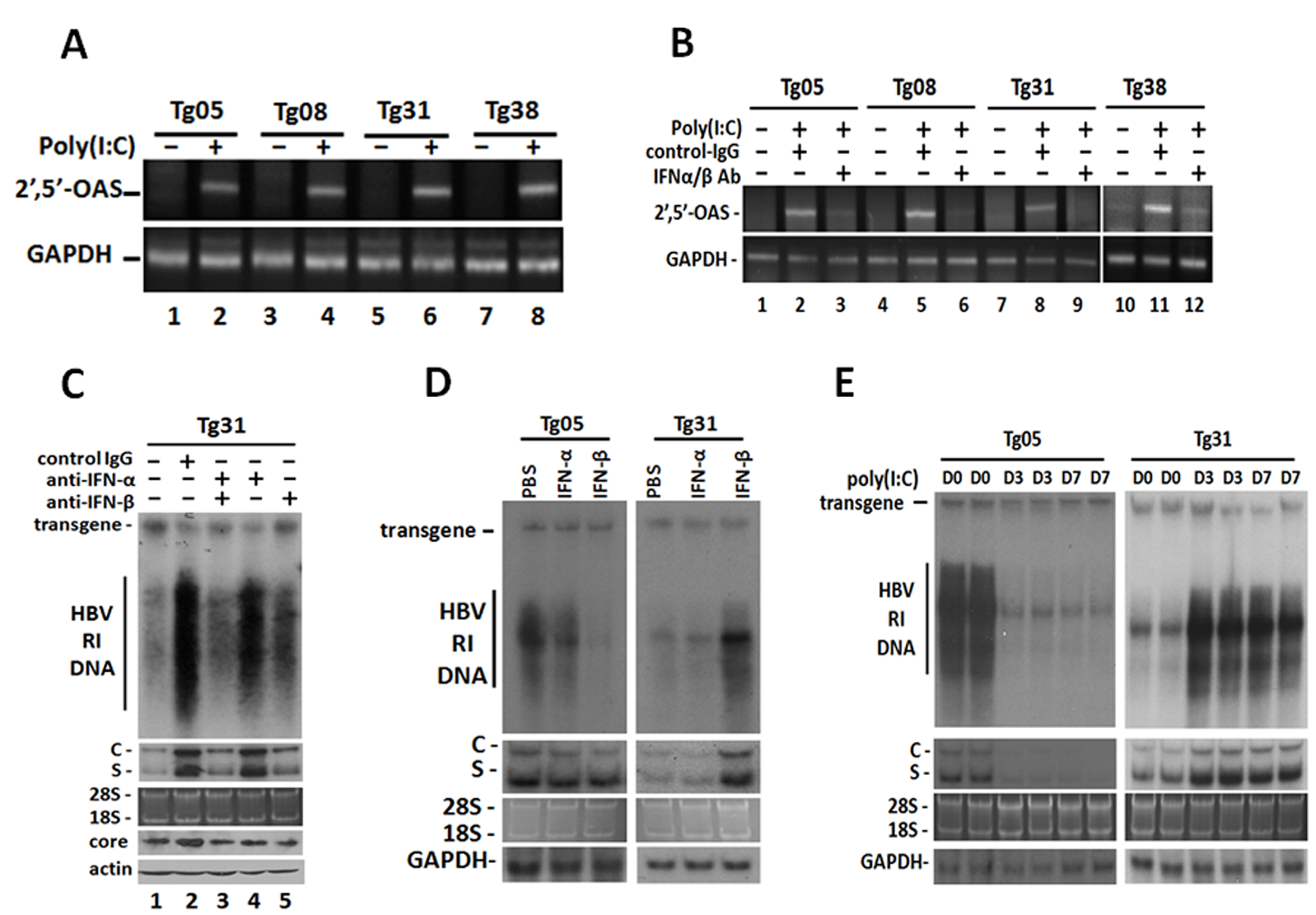

Supplement: Figure S2 — Effects of poly(I∶C) on HBV transgenic mice. (A) Induction of 2′,5′-OAS in the mouse liver by poly(I∶C). Total liver RNA was isolated from mice 24 hours after injection with saline (−) or poly(I∶C) (+) and analyzed by semi-quantitative RT-PCR for 2′,5′-OAS RNA and GAPDH RNA. The latter served as the control. (B) Suppression of the interferon response in the mouse liver by the anti-IFN-α/β antibodies. HBV transgenic mice were injected intravenously with the control IgG or anti-IFN-α/β antibodies (500 µg/mouse) and then with 200 µg poly(I∶C) 16 hours later. Mice were sacrificed 24 hours after the poly(I∶C) injection for the isolation of total liver RNA, which was used for semi-quantitative RT-PCR for the analysis of 2′,5′-OAS RNA and GAPDH RNA. Mice without the injection of poly(I∶C) and antibodies were also included in the studies to serve as the control. (C) Suppression of poly(I∶C)-induced HBV replication in Tg31 HBV transgenic mice by anti-IFN-α/β antibodies. Tg31 HBV transgenic mice were injected intravenously with the control IgG (lane 2), anti-IFN-α and anti-IFN-β antibodies together (lane 3), the anti-IFN-α antibody alone (lane 4), or the anti-IFN-β antibody alone (lane 5), followed by the injection with 200 µg poly(I∶C) 16 hours later (lanes 2–5). Mice were sacrificed 24 hours after the injection with poly(I∶C) for the analysis of HBV DNA (top panel), HBV RNA (middle two panels), and the core protein and β-actin (bottom two panels) in the liver. (D) Effects of IFN-α/β on HBV replication in Tg05 and Tg31 mice. Tg05 and Tg08 mice were injected with PBS, IFN- (1.4×10^5^ units) or IFN-β (1.6×10^5^ units) and sacrificed 24 hours later for the isolation of liver for analysis RNA (second panel from the top). The ribosomal RNAs (third panel from the top) and GAPDH RNA (bottom panel) were used as the loading control for Northern-blot analysis. (E) Prolonged effect of poly(I∶C) on HBV replication. Tg05 and Tg08 mice were injected with poly(I∶C) on a daily basis and sacrifi [file ppat.1002159.s002.tif]

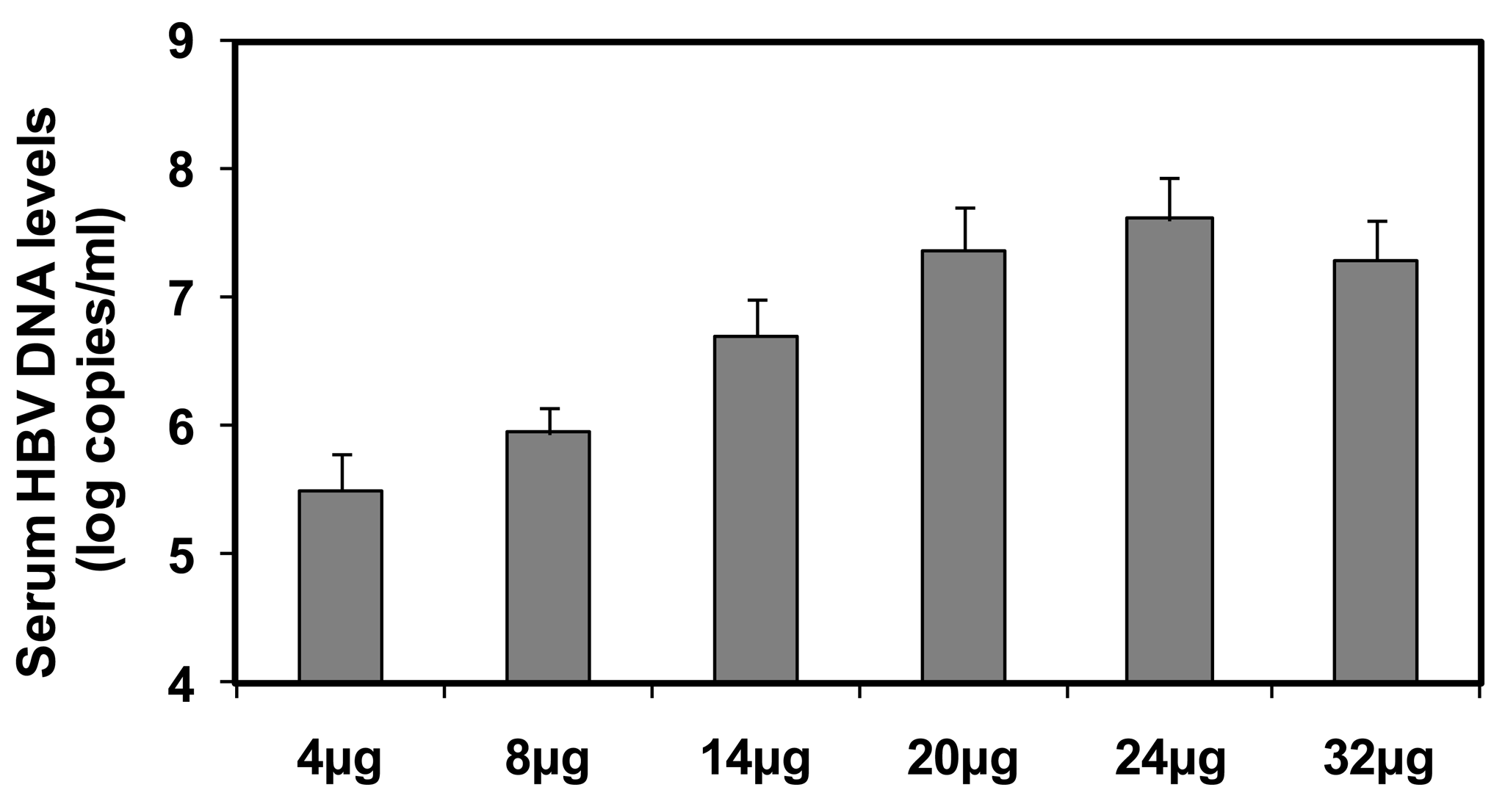

Supplement: Figure S3 — Quantification of HBV titers in the sera of mice injected with HBV DNA. Mice were injected with the indicated amount of the 1.3mer HBV DNA in phosphate-buffered saline (PBS). Four days later, mouse sera were treated with DNase I and micrococcal nuclease to remove free DNA. The HBV virion-associated DNA was then extracted and analyzed by real-time PCR. The results represent the average of at least three different mice. (TIF) [file ppat.1002159.s003.tif]

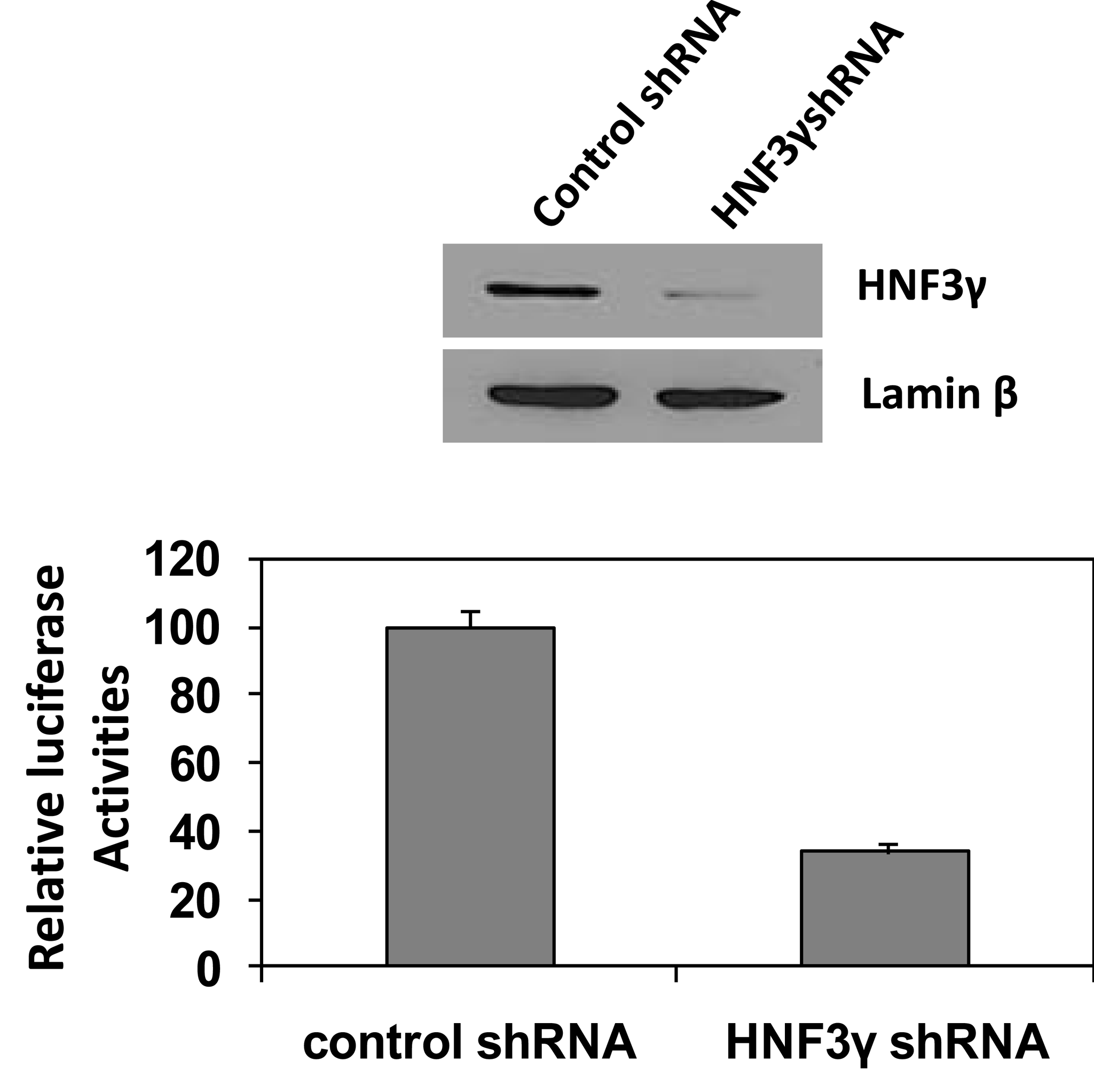

Supplement: Figure S4 — Effects of HNF3γ on the ENI/Xp activity in Huh7 cells. Huh7 cells were co-transfected with the ENI/Xp reporter construct and the expression plasmid for the control shRNA or the HNF3γ shRNA for forty-eight hours and then lysed for Western-blot analysis for HNF3γ and lamin-β (top panel). The latter is a nuclear protein and served as the loading control. The plasmid pRL-SV40, which expresses renilla luciferase, was included in the transfection to monitor the transfection efficiency. Cell lysates were analyzed for the luciferase activities using the dual luciferase assay (Promega) (bottom panel). (TIF) [file ppat.1002159.s004.tif]

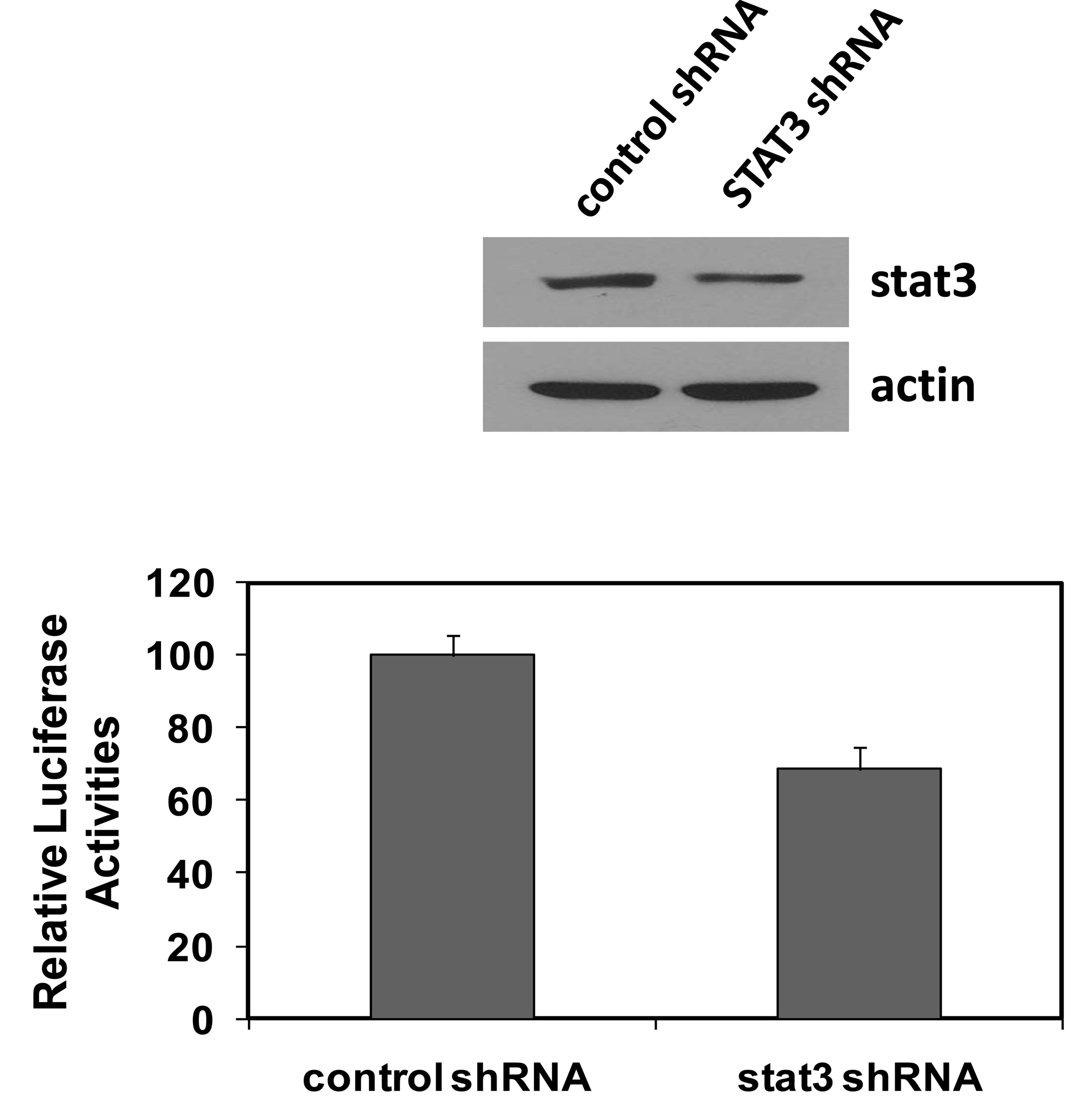

Supplement: Figure S5 — Effects of STAT3 on the ENI/Xp activity in Huh7 cells. The experiments were conducted as described in the legend to Figure S4, with the exception that the HNF3γ shRNA was replaced with the STAT3 shRNA. Top panel, Western-blot analysis of STAT3 and α-actin; bottom panel, relative firefly luciferase activities. (TIF) [file ppat.1002159.s005.tif]

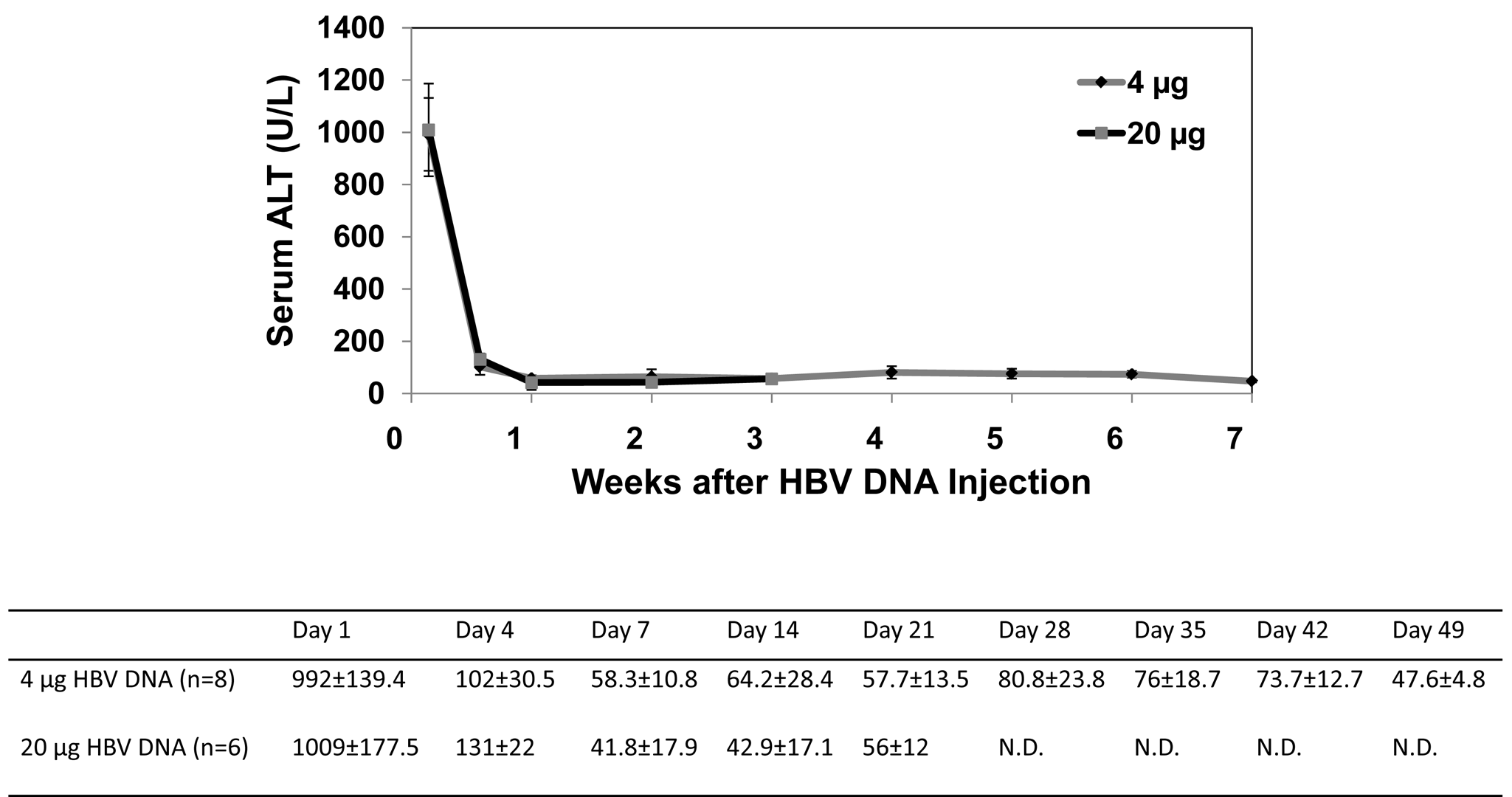

Supplement: Figure S6 — Analysis of ALT levels in mice injected with HBV DNA. The serum samples collected from mice shown in Fig. 6 were analyzed for the ALT levels using the ELISA kit. The numerical ALT levels at individual time points are shown in the Table. N.D., not determined. (TIF) [file ppat.1002159.s006.tif]
